# Imaging in head and neck cancer: United Kingdom National Multidisciplinary Guidelines

**DOI:** 10.1017/S0022215116000396

**Published:** 2016-05

**Authors:** H Lewis-Jones, S Colley, D Gibson

**Affiliations:** 1 Department of Radiology, University Hospital Aintree, Liverpool, UK; 2 Department of Radiology, University Hospital Birmingham NHS Trust, Birmingham, UK; 3 Fiona Stanley Hospital, Murdoch, Perth, WA, Australia

## Abstract

This guideline is endorsed by the specialty associations involved in the care of head and neck cancer patients in the UK. This paper summarises the current imaging modalities in use for head and neck cancer evaluation. It highlights their role in the management with recommendations on modality choice for each cancer subsite.

**Recommendations:**

• Offer appropriate radiological imaging, based on tumour extent, site and local expertise, to stage tumours and plan treatment for patients diagnosed with head and neck cancer. (G)

• Consider positron emission tomography combined with computed tomography (PET–CT) imaging if conventional cross-sectional imaging identifies no primary site. (R)

• Offer PET–CT imaging 12 weeks after non-surgical treatment to detect residual disease. (R)

## Introduction

Imaging in head and neck cancer has developed enormously over the last few decades. Advanced cross-sectional imaging modalities allow accurate staging of disease and contribute significantly to management decisions and prognosis. As a core member of a multidisciplinary team, the radiologist has a key role in presenting relevant multi-modality findings that define disease extent, help with surveillance and highlight pertinent co-morbidities.^1^ This approach also aids pre-treatment counselling and patient consent.

Prior to imaging, the primary site and the presence or absence of neck metastases of a head and neck cancer has often been established clinically and it is not unusual for a histological diagnosis to have been secured from a representative biopsy. Therefore, the primary role of radiology is in accurately staging the full extent and distant spread of disease with the current tumour–node–metastasis (TNM) system, with an emphasis on features that will influence surgical or non-surgical treatment options.

The areas that radiological assessment should focus on are:

• Local extent of the primary tumour
• Spread to locoregional cervical lymph nodes
• Detection of metastatic disease precluding cure and synchronous primary tumours of the lung and upper aero-digestive tract.

## Imaging modalities

### Computed tomography (CT)

Contrast-enhanced CT is the mainstay for imaging primary disease. It is widely available and established in practice. It incurs a significant radiation penalty and iodinated contrast medium is contraindicated in those with severe renal impairment. Conventionally, centres would image the neck and chest at presentation from the skull base to below the diaphragm.

Spatially good but at a radiation cost, CT provides limited soft tissue resolution. Bone detail such as with mandibular or skull base involvement is a major strength. Modern multislice, slip-ring CT detector technology rapidly acquires images without movement artefact as potential head and neck cancer patients may have difficulty with breathing, swallowing secretions and lying flat. Multiplanar and volume rendered images are easily reconstructed. Contrast-enhanced CT allows opacification of vascular structures whilst tumours generally tend to be slower to enhance with a reduced wash out.

### Magnetic resonance imaging (MRI)

Magnetic resonance imaging reflects biochemical tissue characteristics and is largely influenced by proton density and other in situ paramagnetic substances such as blood products and melanin content. Alongside the permanent bore magnet, additional transient magnetic gradients allow the development of an ever increasing array of sequences that are able to reflect pathological processes from normal surrounding tissues. Multiple manufacturers may have differing terms for sometimes similar sequence parameters.

T1-weighted ‘anatomical’ images have excellent spatial resolution, whilst T2-weighted images preferentially highlight oedema and therefore pathology. A short tau inversion-recovery (STIR) sequence retains the positive attributes of a T2-weighted image and suppresses surrounding fat signal in normal or invaded tissues to best depict abnormal tissue as a bright, high signal. Magnetic resonance imaging has the ability to dramatically improve tissue contrast resolution when compared with CT and, in compliant patients without contraindications, it is the imaging modality of choice for defining the primary extent of oral and oropharyngeal cancers. Detrimentally, when compared with CT, scan times are much longer and can vary from about 2–10 minutes for each sequence, during which the patient must keep relatively still. Intravenous gadolinium contrast agents allow static and dynamic vascular assessments of a tumour and, when combined with fat suppression techniques, this can increase the conspicuity of occult pathology. Dental amalgam can reduce the image quality both for CT and MR imaging that makes interpretation more challenging.

### Positron emission tomography combined with CT (PET–CT)

Positron emission tomography combined with CT whole-body imaging uses various labelled tracers to fuse conventional, anatomical CT images with a functional ‘map’ of the disease process. This is conducted on a single gantry at a single appointment. The commonest tracer is 18 fluoro-deoxyglucose, which is preferentially transported and trapped into hypermetabolic cancerous or inflamed tissues. It is detected with a gamma camera array. The patient's fasted baseline glucose level should be measured and the isotope is injected intravenously approximately 1 hour before imaging. The patient refrains from talking or chewing. Actual image acquisition takes about 30–45 minutes. Modern scanner design accurately co-registers metabolic tissue activity with its precise anatomical location.

In 2013, the Royal College of Radiologists published evidence-based guidelines for PET–CT use in head and neck cancer. Evaluating the patient with malignant cervical adenopathy from an unknown primary is one of the main, up-front indications. Positron emission tomography  will detect an occult primary in approximately one third of cases. Positron emission tomography combined with CT is also valuable in the assessment of suspected recurrence of head and neck cancer when there are extensive, confounding post-treatment changes on conventional imaging modalities. Its added benefit in routine surveillance following treatment is still being assessed. Along with other modalities, it has a role in staging malignant thyroid disease including medullary thyroid carcinoma.

### Ultrasound

Offered as part of a modern one-stop service, ultrasound, alongside fine needle aspiration cytology, allows rapid imaging assessments for those with an undiagnosed neck lump or suspected metastatic disease in the neck. This technique can be notoriously operator dependent, but has no detrimental patient effects. Following slide preparation, best cytological practice recommends prompt adequacy assessments and, ideally, the cytologist should be onsite for diagnostic advice. In reality, a shortage of radiology and histopathological input makes such universal service developments difficult.

Ultrasonography comfortably delineates thyroid pathology and can detect occult pathological nodes (necrosis, microcalcification, etc.) that may feel clinically normal in size. A normal node should remain ovoid in shape with a short axis diameter less than 10 mm with a preserved echogenic hilum. Retropharyngeal and superior mediastinal nodes cannot be assessed with this modality.

Current doctrine dictates that clinically and radiologically N0 disease from high-risk primary sites is presumed to have small volume nodal micrometastasis that routinely requires prophylactic first-line treatment as no available tests can guarantee a true pathological N0 status.

### Fluoroscopy

There are a variety of scenarios when contrast swallows and fluoroscopy are used in head and neck cancer, although the availability of local expertise can be variable. Contrast swallows can be used to assess the length of a malignant proximal oesophageal stricture, while the risk of airway aspiration or penetration is dynamically assessed by videofluoroscopy. Alternative, non-oncological causes for dysphagia such as a pharyngeal pouch may be diagnosed. Water soluble contrast studies are advised when the risk of aspiration is high, for instance, following recurrent chest infections or diminished pharyngeal sensory/motor function after surgery or radiation. The integrity of a surgical anastomosis or the tract of an entero-cutaneous fistula can also be well evaluated. These studies are often jointly performed with a speech and language therapist to facilitate decision making and may improve functional outcomes.

### Chest imaging

With common aetiological factors, patients with head and neck cancer have higher incidences of synchronous and metachronous primary lung tumours that may be disseminated at presentation. At staging, CT imaging of the thorax is routinely advised.

The most common protocol for patients with a head and neck cancer will therefore be to image the primary site by either contrast-enhanced CT or magnetic resonance imaging (MRI), perform CT imaging of the chest and PET–CT for the unknown primaries.

## Specific tumour sites

This section deals with specific tumour sites and highlights areas where radiological evaluation is particularly important and often difficult.

### Oral cavity

Preferred imaging modality: MRI

Tongue tumours are routinely evaluated with MRI to aid treatment choices and prognosis. Early or advanced cancers of the buccal mucosa, retromolar trigone, palatal and floor of mouth are more difficult to evaluate reliably by imaging alone and good clinical correlation is essential. Perineural and marrow involvement is best defined at MRI.

In an attempt to avoid osteoradionecrosis, orthopantomograms are still requested to proactively treat dental caries and peri-apical disease.

### Oropharynx

Preferred imaging modality: MRI

Small or subclinical primaries in the tonsil and tongue base that often present with cervical lymphadenopathy can be difficult to identify with all forms of imaging including PET–CT. These tumours are often best evaluated at MRI with STIR sequences and often, may only be localised retrospectively after examination and biopsy under anaesthesia. Extension of mucosal tumours into the adjacent structures and neck spaces is well depicted with MR imaging.

### Nasopharynx

Preferred imaging modality: MRI

Nasopharyngeal tumours commonly present at an advanced stage with palpable nodal neck disease. Magnetic resonance imaging allows accurate classification of the primary site and nodal disease as per the TNM classification, based on disease extent.

### Hypopharynx

Preferred imaging modality: MRI

In those patients who have difficulty with swallowing, aspiration or breathing when supine, a CT scan will need to be strongly considered.

### Larynx

Preferred imaging modality: MRI

Disease at the level of the vocal cords presents early with dysphonia and is well localised. Imaging is often unnecessary for T1 disease unless extralaryngeal disease, cartilage involvement, nodal metastasis or chest pathology is suspected. MRI with contrast is the gold standard for depiction of cartilage involvement.

**Recommendations**

• Offer appropriate radiological imaging, based on tumour extent, site and local expertise, to stage tumours and plan treatment for patients diagnosed with head and neck cancer (G)
• Consider PET–CT imaging if conventional cross-sectional imaging identifies no primary site (R)
• Offer PET–CT imaging 12 weeks after non-surgical treatment to detect residual disease (R)

### Salivary glands

Malignant salivary glands neoplasms are a very heterogenous group of tumours, where tumour behaviour and prognosis is dictated by the histology. Ultrasound techniques have a significant role to play in assessing the parenchymal mass, local adenopathy and guiding biopsies. Perineural or skull base involvement often requires a combined multi-modality CT and MR approach. The best imaging modality may be guided by site-specific characteristics such as respiratory motion artefact.

### Sinuses

MRI with contrast is the modality of choice to assess surgical resectability issues around intracranial and orbital disease spread. Skull base involvement usually requires a complementary CT study.

## Post-operative imaging

The choice of imaging in the post-operative scenario is determined by the specific clinical question posed. Complications are frequent with difficult head and neck resections. When the specific question is over potential residual or recurrent disease, following either surgery or chemoradiotherapy, the choice for baseline imaging mainly falls between a contrast CT of the neck and chest and a timely PET–CT study. As an exception, MRI has a large role to play specifically for nasopharyngeal, sinonasal and skull base tumour follow up.^2^ Early detection of residual disease is vital to planning further curative attempts. The timing of the scan is important. Dedicated CT gives better resolution and anatomical detail at the primary site as well as detecting subcentrimetre early metastatic disease in the lungs. Obliteration of fat planes and anatomical distortions makes interpretation difficult. A negative, normal PET–CT 12 weeks post-treatment likely offers the best prognostic reassurance currently.^3^ PET–CT fails to reliably distinguish inflammatory elements from malignant foci. Ultrasound guided procedures still have a role to play in sampling indeterminate, persistent enlarged cervical nodes.

### Key points

• Accurate image interpretation and staging heavily influences optimal treatment strategies
• Contrast-enhanced computed tomography of the skull base, neck and chest is ubiquitous in nature, readily available and the workhorse for routine tumour–node–metastasis staging of head and neck cancers
• Positron emission tomography combined with computed tomography is of proven diagnostic benefit when searching for the unknown primaries, when conventional imaging is non-informative
• In compatible patients, magnetic resonance imaging has superior soft tissue characterisation at several primary sites including oropharynx, nasopharynx/skull base and sinuses that greatly aid surgical planning and resections
• Ultrasound image guided diagnostic fine needle and core biopsies are well established and cost-effective in the context of good cytological/histological support
• In certain instances, multi-modality approaches are complementary to each other but should not adversely impact on the speed of the diagnostic pathway.
